# Movement Disorders in Neurocysticercosis: A Systematic Review

**DOI:** 10.5334/tohm.1061

**Published:** 2025-08-08

**Authors:** Ravindra Kumar Garg, Shweta Pandey, Apoorva Agrawal, Raza Abbas Mahdi, Sanjay Singhal

**Affiliations:** 1 Department of Neurology, Era’s Lucknow Medical College & Hospital, Era University, Lucknow, India; 2 Department of Neurology, King George’s Medical University, Lucknow, India; 3 Department of Pathology, Era’s Lucknow Medical College & Hospital, Era University, Lucknow, India; 4 Department of Nuclear Medicine, Postgraduate Institute of Medical Education and Research, Chandigarh; 5 Department of Pulmonary Medicine, Dr. Ram Manohar Lohia Institute of Medical Sciences, Lucknow, India

**Keywords:** Cysticercosis, Movement Disorders, Ataxia, Dystonia, Taenia solium, Tremors

## Abstract

**Background:**

Neurocysticercosis, caused by *Taenia solium* larvae, is a common parasitic infection of the central nervous system in endemic regions. Although seizures and headaches are the typical presentations, movement disorders represent a lesser-known but clinically important manifestation. This systematic review aimed to examine the spectrum, radiological correlates, treatments, and outcomes of movement disorders associated with neurocysticercosis.

**Methods:**

A comprehensive search of PubMed, Scopus, Embase, Web of Science, and Google Scholar was conducted to identify case reports, case series, and cohort studies reporting movement disorders in confirmed cases of neurocysticercosis. Included studies were assessed for clinical details, neuroimaging findings, cerebrospinal fluid results, type of movement disorder, treatment regimens, and outcomes. Quality assessment was done using Murad’s framework for case reports.

**Results:**

A total of 45 patients were included: 21 presented with hypokinetic and 24 with hyperkinetic movement disorders. In the hypokinetic group, parkinsonism was the most common manifestation (52.38%) with basal ganglia and midbrain involvement. Levodopa was administered in 28.57%, and 47.62% achieved marked clinical improvement. In the hyperkinetic group, chorea (29.17%), facial dyskinesias (25%), and myoclonus (16.67%) were prevalent. Albendazole with corticosteroids was used in over 60% of cases, and 83.3% had full or marked recovery.

**Conclusions:**

Movement disorders in neurocysticercosis are diverse but potentially reversible. Early neuroimaging and targeted therapy yield favorable outcomes, especially in hyperkinetic presentations.

## Introduction

Neurocysticercosis, resulting from infection with the larval stage of *Taenia solium*, is a major cause of seizures globally, especially in low-income areas where pig farming is prevalent. Clinically, it presents with seizures, headache, intracranial hypertension, focal deficits, or hydrocephalus, depending on cyst location. Though non-endemic in countries like the USA, immigration leads to significant emergency visits and neurological consultations [1].

Parasitic infections of the central nervous system can result in a range of movement disorders due to inflammation, granuloma formation, or mass effects on motor control regions such as the basal ganglia, brainstem, and cortex. Cerebral malaria, a common cause, is associated with Parkinsonism, dystonia, chorea, ataxia, and spasticity. Toxoplasmosis, particularly in immunocompromised individuals, may cause Parkinsonism and chorea. Rarely, African trypanosomiasis can present with tremors, choreiform or athetoid movements, dysarthria and cerebellar ataxia. In rare cases, echinococcosis (hydatid disease) may cause dyskinetic or pseudoathetotic movements due to mass effect from cysts in motor pathways [2,3,4,5,6].

Neurocysticercosis, caused by the larval form of *Taenia solium*, is the most common parasitic infection of the central nervous system and a leading cause of acquired epilepsy in endemic regions [1]. Although seizures are the predominant clinical manifestation, a diverse spectrum of movement disorders has also been reported. These include parkinsonism, tremor, dystonia, chorea, ataxia, myoclonus, tics, and hemifacial spasm. Movement disorders in neurocysticercosis may arise from lesions in the basal ganglia, thalamus, cerebellum, or brainstem, or secondary to complications such as hydrocephalus [7,8,9].

Despite scattered case reports and small series documenting these associations, the true burden, spectrum, anatomical correlations, and therapeutic responses of movement disorders in neurocysticercosis remain poorly characterized. The objectives of this review are to describe the types and frequency of movement disorders seen in patients with neurocysticercosis, to identify the clinical and radiological features associated with these disorders, to summarize the treatment approaches used for both the parasitic infection and the movement symptoms, to assess the treatment outcomes and long-term responses, and to explore the proposed pathogenetic mechanisms that may explain the occurrence of these movement disorders in the context of neurocysticercosis.

## Methods

This systematic review was carried out following the guidelines of the Preferred Reporting Items for Systematic Reviews and Meta-Analyses (PRISMA).The methodology was designed to comprehensively identify, select, and synthesize published literature on movement disorders associated with neurocysticercosis, including case reports, case series, and observational cohort studies. The review protocol was developed a priori and includes detailed eligibility criteria, search strategy, study selection process, data extraction methods, quality assessment framework, and synthesis approach. The protocol was registered with PROSPERO (CRD420251065860) [10].

## Eligibility Criteria

Reports were eligible for inclusion if they fulfilled the diagnostic criteria of neurocysticercosis escribed by Del Brutto and colleagues [11]. Subtypes of movement disorders were categorized based on the definitions established by the Movement Disorder Society [12].

Reports were not considered if the diagnosis of neurocysticercosis or the movement disorder was not clearly established, if the report involved animal models, or if the movement disorder was due to other coexisting pathologies not attributed to neurocysticercosis.

## Search Strategy

A comprehensive literature search was conducted using PubMed, EMBASE, Scopus, and the first 50 pages of Google Scholar. Boolean operators were applied, and the search strategy was customized for each database by incorporating appropriate controlled vocabulary (e.g., MeSH in PubMed and Emtree in EMBASE). The search string used was: ((“Neurocysticercosis”[Mesh] OR neurocysticercosis OR NCC OR “cysticercosis of brain” OR “cysticercosis of central nervous system” OR “cysticercus cellulose”) AND (“Movement Disorders”[Mesh] OR “movement disorder*” OR tremor OR dystonia OR chorea OR myoclonus OR parkinsonism OR “extrapyramidal symptoms” OR “involuntary movements” OR “dyskinesia” OR tics). The final search was completed on June 2, 2025. No restrictions were placed on publication date or language.

## Study Selection

Data collection was conducted in two sequential stages. Initially, relevant studies were identified and screened. This was followed by a detailed full-text review using predefined inclusion and exclusion criteria, carried out by RKG and SP. Any discrepancies were resolved through consultation with a third reviewer (RAM). Duplicate entries were removed using EndNote 21 (Clarivate Analytics, Philadelphia, USA). A PRISMA flow diagram was constructed to depict the study selection process. Extracted variables included patient demographics, clinical features, neuroimaging findings, treatment approaches, and outcomes. Data extraction was independently performed by three reviewers (AA, RAM, and SS), with any disagreements resolved by a fourth reviewer (RKG).

## Data Extraction

The variables extracted included first author and year of publication, country of origin, patient age and sex, duration of illness, type of movement disorder, type and location of neurocysticercosis on neuroimaging, other neurological manifestations, cerebrospinal fluid findings if available, antiparasitic and steroid therapy administered, treatment specifically for the movement disorder, radiological and clinical outcomes, follow-up duration, and the proposed pathogenetic mechanism of the movement disorder. If multiple patients were described in a single report, data were extracted for each patient individually.

## Quality Assessment

The quality of the included studies was evaluated using the framework developed by Murad et al. for the critical appraisal of case reports and case series [13]. This framework examines four domains: (1) selection of patients, (2) ascertainment of diagnosis, (3) demonstration of causality, and (4) reporting detail. Each study was rated as “good,” “fair,” or “poor” based on how many criteria were satisfied [14]. Two reviewers (RKG and SP) independently conducted the quality assessment, and any discrepancies were resolved through consensus.

## Data Synthesis

Given the heterogeneity in study designs, clinical presentations, and outcome measures, a narrative synthesis was conducted. Frequencies and proportions were calculated for categorical variables such as types of movement disorders, anatomical sites of lesions, and treatment modalities. Descriptive statistics were used for continuous variables. Clinical patterns, imaging correlates, and outcomes were summarized in structured tables. Where appropriate, subgroup analyses were performed based on age groups, lesion locations, or type of movement disorder.

## Results

**Figure 1:**
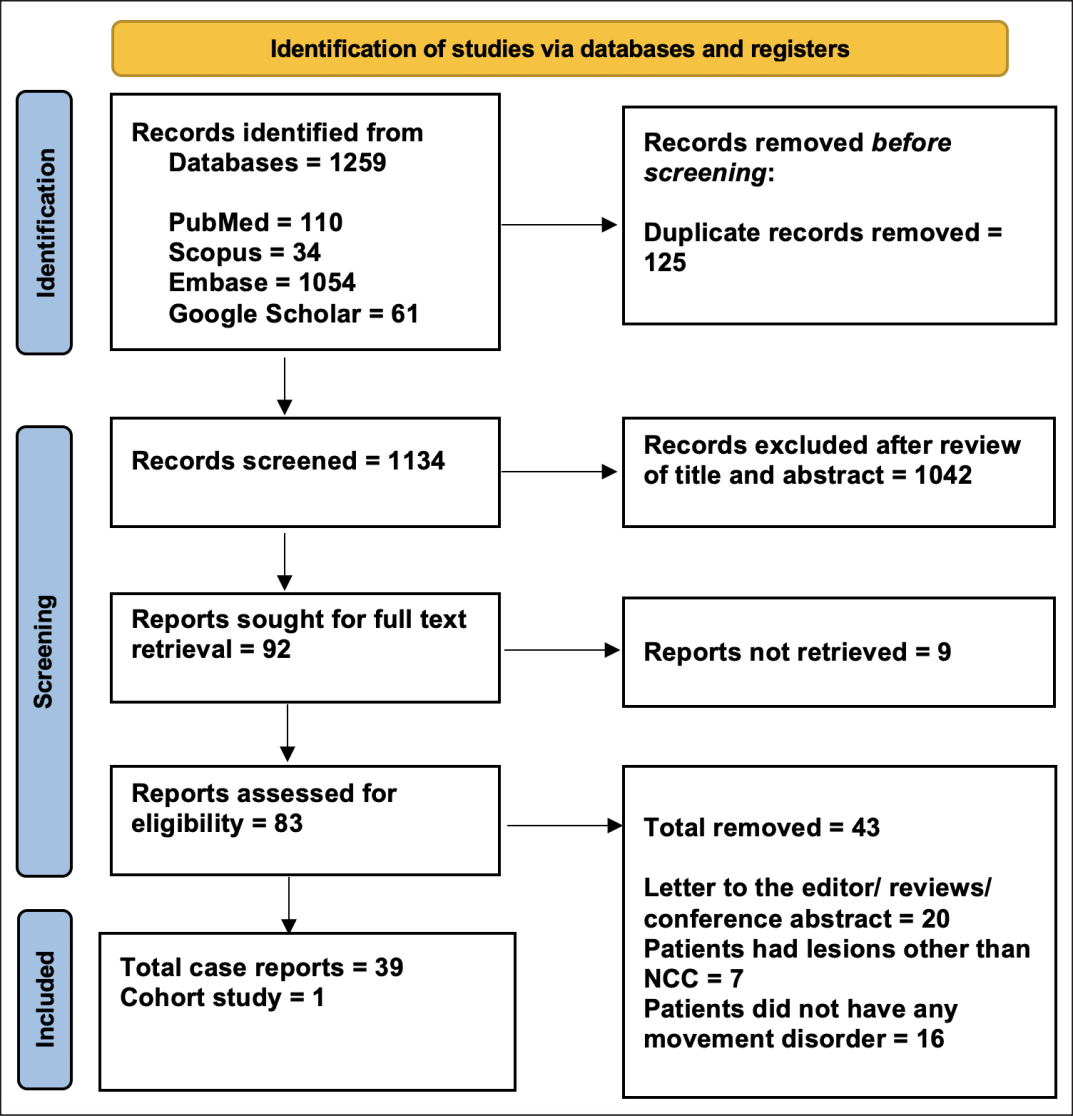
The PRISMA flow diagram illustrates the study selection process for the systematic review.

This review included 45 patients with neurocysticercosis and associated movement disorders, of which 21 had hypokinetic and 24 dominantly had hyperkinetic manifestations [15,16,17,18,19,20,21,22,23,24,25,26,27,28,29,30,31,32,33,34,35,36,37,38,39,40,41,42,43,44,45,46,47,48,49,50,51,52,53]. (Tables 1 and 2) All individual cases were rated as good quality (Supplementary Item 1). The PRISMA checklist is provided as Supplementary Item 2. Figure 1 depicts the PRISMA 2020 flow diagram summarizing the process of study identification, screening, eligibility assessment, and final inclusion in the systematic review.

**Table 1:** Hypokinetic Movement Disorders in Neurocysticercosis: A Summary of Reported Cases (n = 21).


REFERENCE	COUNTRY	AGE/ SEX	DURATION OF ILLNESS	TYPE OF MOVEMENT DISORDER	TYPE OF NEURO CYSTI CERCOSIS/ NEU ROIMAGING	OTHER NEU ROLOGICAL FINDINGS	CSF FINDINGS	TREATMENT GIVEN ANTIPA RASITIC/STEROIDS)	TREATMENT OF MOVEMENT DISORDER	RESPONSE TO TREATMENT	FOLLOW-UP NEU ROIMAGING, IF ANY	OUTCOME	POSSIBLE REASONS OF MOVEMENT DISORDERS

Das et al 2025 [15]	India	23/M	7 days	Mutism Echolalia Gegenhalten (resistance to passive movement) Cataplexy (sudden loss of muscle tone) Abnormal limb posturing	Multiple NCC in different stages	Seizure history, reduced interaction, immobility	Not mentioned	NA	Lorazepam 12 mg/day, Amantadine 400 mg/day	Marked improvement from day 1, complete recovery in 4 weeks	Not reported	Complete recovery	Diffuse cortical and basal ganglia involvement from NCC; catatonia mechanism involving cortico-subcortical motor circuits

Rasaholiarison et al 2024 [16]	Madagascar	11/F	4 years (onset at 7 years)	Dystonic tremor of the left upper limb Kinesigenic dystonia of the left lower limb	A ring lesion in right thalamus with perilesional edema	Left hemiparesis Amyotrophy (muscle wasting) Walking disorder Enuresis	Not reported	Albendazole (8 mg/kg/day × 15 days), corticosteroids (1 mg/kg/day × 5 days)	Tropatepine 20 mg/day, physiotherapy	Lesion improved radiologically; movement disorder persisted	CT showed calcification (sequela of thalamic NCC)	Partial improvement; persistent movement disorder	Right thalamic NCC affecting cerebello-thalamo-cortical pathways; inflammation and sequelae

Ahmad N et al 2024 [17]	India	6/M	7 months	Painful, sustained tightening of all four limbs (generalized dystonia)	Multiple cystic lesions with scolex (basal ganglia, cortical/subcortical areas, thalamus)	Normal	CSF anticysticercal antibody strongly positive	Albendazole	Not specifically mentioned	Significant clinical and radiological improvement after 6 weeks	Yes, showing improvement	Improved	Inflammatory reaction around cysts; basal ganglia involvement; perilesional edema

Puig et al 2023 [18]	Spain	49/M	2 days (onset of dystonic tremor); history of seizures years prior	Dystonic head tremor; left faciobrachial clonic movements (partial seizures)	Multiple NCC lesions; one in subthalamic nucleus and red nucleus	Normal	Not reported	Anthelmintic treatment considered but deferred due to risk of inflammatory response	Botulinum toxin injections; beta-blockers and primidone ineffective	Partial improvement with botulinum toxin	Not specified	Partial improvement	Lesions in subthalamic/red nucleus and cerebello-thalamo-cortical pathway involvement

Oliveira et al 2020 [19]	Brazil	59/F	4 months	Parkinsonism (bradykinesia, stiffness, gait disturbance)	Parenchymal and intraventricular NCC; hydrocephalus; MRI: cysts with scolex, calcifications, transependymal edema	Urinary incontinence, dementia, epilepsy (history of 15 years)	Normal CSF findings	Albendazole and praziquantel (14 days); corticosteroids used initially	Ventriculoperitoneal shunt (VPS); symptomatic therapy as needed	Clinical improvement after VPS	Post-op CT showed well-placed drain and calcifications	Marked improvement in parkinsonian symptoms	Hydrocephalus-induced compression of nigrostriatal pathway; mass effect and inflammation from cysticerci

Verma R et al 2013 [20]	India	50/F	3 months	Parkinsonism	MRI: Multiple cystic lesions with mural nodules in bilateral basal ganglia	Hypokinetic speech, slow ocular movements, reduced blink, positive Meyerson’s sign, generalized rigidity, severe bradykinesia, postural instability	Not reported	Albendazole 15 mg/kg/day with steroids	Not separately given	Deteriorated with worsening sensorium; died despite anti-edema measures	Not reported	Death	Inflammatory reaction due to multiple basal ganglia cysts disrupting dopaminergic pathways

de Lima PMG et al 2012 [21]	Brazil	38/F	4 months	Parkinsonism (rigidity, bradykinesia, tremor, postural instability)	MRI: Intraventricular cyst at frontal horn of right lateral ventricle extending through foramen of Monro to third ventricle; edema in midbrain periaqueductal region	Mutism, hypomimia, sialorrhea, anarthria, vertical ophthalmoparesis, severe intracranial hypertension	Positive for antibodies in CSF	Albendazole (3.2 g/day), later methylprednisolone 1 g IV x5 days	Levodopa/carbidopa (250/50 mg daily)	Progressive improvement, remained asymptomatic after 8 months	MRI showed stable cyst and edema initially; post-surgical changes not specified	Full recovery, returned to routine activities	Hydrocephalus, ependymitis, and inflammatory edema affecting dopaminergic midbrain pathways

Sharma et al 2011 [22]	India	64/M	1.5 years	Parkinsonism Progressive supranuclear palsy (PSP)-like syndrome	MRI: Multiple vesicular NCC lesions in dorsal and tegmental midbrain, also in bilateral cerebral hemispheres	Hypophonic spastic speech, MMSE 16, vertical gaze palsy (up-gaze > down), slow horizontal saccades, axial rigidity, brisk DTRs, primitive reflexes, cognitive decline	Positive ELISA for NCC, negative for HIV; other CSF details not reported	Albendazole for 2 weeks + dexamethasone	Not specifically treated; only antiparasitic and steroids given	No significant clinical improvement after 2 months	Not performed	Persistent disability	Midbrain involvement by NCC lesions affecting vertical gaze and extrapyramidal pathways, mimicking PSP

Prashantha et al 2008 [23]	India	38/M	1 year with acute onset after 2nd VP shunt	Bilateral Parkinsonism with resting and postural tremor (5–5.5 Hz), bradykinesia, rigidity, hypomimia	MRI: Enhancing granuloma with scolex in fourth ventricle, significant perilesional edema involving cerebellum	No cognitive or ocular deficits; rigidity, bradykinesia, mild dysarthria, normal reflexes	Normal CSF during 1st shunt; no repeat CSF after onset	Short course of steroids (due to perilesional edema); no antiparasitic given	Levodopa (275 mg × 4/day) + Trihexyphenidyl (up to 12 mg/day)	Slow response initially; marked clinical improvement by 3 weeks; asymptomatic off drugs by 3 months	Follow-up MRI: Lesion persisted but edema decreased; no hydrocephalus	Near-complete recovery without recurrence	VP shunt may have altered CSF dynamics triggering basal ganglia dysfunction; possible transient inflammation or degeneration of cyst; not direct NCC effect

Cabo López et al 2008 [24]	Spain	29/F	Not precisely stated (chronic course with hydrocephalus)	Parkinsonism (L-dopa responsive)	Cyst in the fourth ventricle causing obstructive hydrocephalus	Multiple episodes of shunt malfunction; no focal deficits described	Not detailed	Cysticidal drugs and repeated shunt surgeries	L-dopa	Marked improvement with L-dopa and antiparasitic therapy	Not reported	Improved; symptoms resolved	Raised intracranial pressure from obstructive hydrocephalus due to IV ventricle cyst affecting midbrain basal ganglia circuits

Patel et al 2006 [25]	India	60/M	Not clearly stated	Parkinsonism, dystonia, tremor, gait disturbance	Multiple cysticerci (vesicular and granular) including basal ganglia	Normal cognition (MMSE), no cerebellar signs	Positive ELISA, normal biochemistry and cytology	None specifically mentioned	Levodopa + Trihexyphenidyl	Initial improvement, lost to follow-up	Not mentioned	Initial improvement, lost to follow-up	Basal ganglia involvement (caudate, putamen, thalamus)

		19/F	Not clearly stated	Focal dystonia (left upper limb)	Single ring-enhancing granuloma in right thalamus (colloidal)	Normal mental and systemic exams	Normal	Albendazole + Steroids	None specific	No improvement, lost to follow-up	Not mentioned	No improvement, lost to follow-up	Thalamic lesion causing focal dystonia

Sá et al 2005 [26]	Brazil	32/F	Rapidly progressive over 1 month	Parkinsonism Symmetrical resting tremor, rigidity, bradykinesia, postural instability	Cysticercotic ependymitis, MRI showed lesions at cerebral aqueduct and 4th ventricle	Vertical gaze paresis, hypomimia, sialorrhea, diffuse hyperreflexia	WBC 112 (60% lymphocytes), protein 91 mg/dL, glucose 45 mg/dL, NCC positive (ELISA, IIF)	Albendazole (30 mg/kg/day × 60 days), steroids not specified	Levodopa 125–250 mg tid	Marked improvement; able to walk unaided; later mild symptoms required levodopa re-initiation	Not specified	Clinically better, residual mild parkinsonism	Midbrain lesion and inflammation affecting substantia nigra

		30/M	2 weeks	Parkinsonism Marked rigidity, bradykinesia, postural instability	MRI: cysticercotic ependymitis affecting quadrigeminal/ambient cisterns; hydrocephalus on CT	Headache, vomiting, reduced consciousness	WBC 81 (64% lymphocytes), protein 90 mg/dL, glucose 42 mg/dL, NCC positive (ELISA, IIF)	Albendazole (high dose), steroids not specified	Levodopa	Improved consciousness, motor function; mild bradykinesia remained at 6 months	MRI abnormalities observed	Sustained clinical improvement at 6 months	Brainstem lesions with inflammation near substantia nigra

Serrano-Dueñas & Placencia 1999 [27]	Ecuador	75/M	8 years	Tremor	Cortical and subcortical cystic lesions, hydrocephalus	Gait disorder, cognitive decline	Positive anticysticercal antibodies	Albendazole 15 mg/kg/day × 8 days	VP shunt replacement	Improved; tremor disappeared by 6 months	Not reported	Tremor resolved	Hydroce phalus and neurocy sticercosis-related basal ganglia involvement

		66/M	10 years	Parkinsonism, Tremor	Obstructive hydrocephalus, fourth ventricle cysticercus, multiple calcifications	Diplopia, headache, rigidity, bradykinesia	Positive anticysticercal antibodies	Albendazole × 8 days, surgical cyst removal, VP shunt	Clonazepam 2 mg/day	Improved after surgery	Not specified	Good recovery maintained over 3 years	Compression from cyst, basal ganglia involvement

		40/F	4 years	Tremor	Third ventricle cysticercus, cortical/subcortical lesions	Headache, visual decline	Positive anticysticercal antibodies	Albendazole × 8 days, Prednisone, VP shunt	None specified	Improved, tremor resolved in 7 days	Lesion resolution	Tremor resolved	Compression and cystic lesions affecting motor areas

		60/M	6 years	Dystonia (retrocollis)	Cortical/subcortical lesions, hydrocephalus	Memory, speech, gait disturbances	35 cells/mm^3^, protein 56 mg/dL, positive antibodies	Albendazole × 8 days, VP shunt	None specified	Gradual improvement, dystonia resolved	Not reported	Complete recovery at 1 year	Brainstem involvement, disinhibition of facial/trigeminal motor nuclei

Sawhney et al 1998 [28]	India	21/M	2 years	Unilateral dystonia (right upper and lower limbs)	Temporal/frontal lobe, hypodense cysts in globus pallidi, ring-enhancing lesion in corona radiata, bifrontal diffuse white matter oedema	Complex partial seizures	Not reported	Steroids, anticonvulsants	Anticonvulsants	Improved	Not reported	Improved	Ictal dystonia due to temporal lobe epilepsy with spread to basal ganglia

Verma et al 1995 [29]	USA	31/F	2 days (parkinsonism developed later during treatment)	Parkinsonism (hemi parkinsonism, rigidity, tremor, bradykinesia)	Enhancing cyst in periaqueductal gray and ventral midbrain; cyst in 4th ventricle; multiple calcifications	Vertical gaze palsy, diplopia, ptosis, corectopia, hemiparesis, stupor, central 7th nerve palsy	Opening pressure 280 mm H2O, 120 cells/µL (98% monocytes), protein 54 mg/dL, glucose 57.7 mg/dL	Praziquantel 50 mg/kg/day × 21 days, IV dexamethasone	Supportive care, corticosteroids; no specific antiparkinsonian drug mentioned	Marked improvement; near-complete resolution of parkinsonism	Not specified in detail post-recovery	Full recovery with residual gaze palsy and corectopia	Inflammatory response to degenerating cyst in midbrain structures (substantia nigra, periaqueductal gray)

De Assis et al 1955 [30]	Brazil	26/F	1.5 months	Parkinsonism	Racemose cyst in interpeduncular fossa compressing brainstem; multiple cysticerci in cerebral hemispheres (autopsy)	Vertical gaze palsy, bilateral facial weakness, extrapyramidal hypertonia, somnolence, papilledema, cranial nerve palsies	CSF: 26 cells/mm^3^ (lymphomononuclear), protein 25 mg/dL, glucose 52 mg/dL, Pandy +; serology negative	Not specified (diagnosis made postmortem)	Not given	No improvement; progressive deterioration	Not applicable (autopsy diagnosis)	Death in coma	Compression of midbrain, subthalamus, substantia nigra, and hypothalamus by racemose cysticercus; inflammatory damage


AFB = Acid-Fast Bacilli; ANA = Antinuclear Antibody; ASO = Anti-Streptolysin O; CSF = Cerebrospinal Fluid; CT = Computed Tomography; DTR = Deep Tendon Reflex; ELISA = Enzyme-Linked Immunosorbent Assay; HIV = Human Immunodeficiency Virus; IV = Intravenous; JE = Japanese Encephalitis; MMSE = Mini-Mental State Examination; MRI = Magnetic Resonance Imaging; NA = Not Available/Not Applicable; NCC = Neurocysticercosis; PSP = Progressive Supranuclear Palsy; SN = Substantia Nigra; TB = Tuberculosis; TB-PCR = Tuberculosis–Polymerase Chain Reaction; VP Shunt/VPS = Ventriculoperitoneal Shunt; WBC = White Blood Cell Count.

**Table 2:** Hyperkinetic Movement Disorders in Neurocysticercosis: A Summary of Reported Cases.


REFERENCE	COUNTRY	AGE/SEX	DURA TION OF ILLNESS	TYPE OF MOVEMENT DISORDER	TYPE OF NEURO CYSTICERCOSIS/ NEUROI MAGING	OTHER NEURO LOGICAL FINDINGS	CSF FINDINGS	TREATMENT GIVEN ANTIPA RASITIC/STEROIDS)	TREATMENT OF MOVEMENT DISORDER	RESPONSE TO TREATMENT	FOLLOW-UP NEUROI MAGING, IF ANY	OUTCOME	POSSIBLE REASONS OF MOVEMENT DISORDERS

Matos Pereira 2022 [31]	Portugal	59/M	1 hour (acute onset of myoclonus)	Right hand myoclonus	Multiple cystic lesions CT: 3 hypodense cystic lesions with hyperdense center MRI: bilateral cysts and right hippocampal nodular lesion with edema	None reported	Not reported	Albendazole and dexamethasone	Phenytoin	Complete resolution of myoclonus	MRI at 3 months showed resolution of lesions	No recurrence of myoclonus at 3 months	Lesions involving motor cortex or hippocampus; inflammation and edema

Yang et al 2020 [32]	Peru	38/M	2 months	Hemifacial spasm (left side)	Subarachnoid NCC; cyst in cerebellopontine angle with severe arachnoiditis	Obstructive hydrocephalus (VP shunt placed 6 months earlier)	Not reported	Albendazole + dexamethasone (no response)	Surgical decompression and cyst excision	Complete resolution of hemifacial spasm	Not reported post-op, but intraoperative imaging confirmed cyst removal	Symptom-free at 8-month follow-up	Compression and irritation of the facial nerve due to arachnoiditis from NCC cyst in cerebellopontine angle

Kumar S et al 2020 [33]	India	77/M	15 days	Hemichorea (right upper limb)	Multiple cysts in cortex, and basal ganglia with surrounding edema and calcified scars on MRI	None besides hemichorea and mild shoulder pain	Not reported	Albendazole 400 mg BID + Prednisolone 1 mg/kg/day	NA	Marked improvement within 1 week	Marked reduction in lesions at 1-month MRI	Significant clinical improvement	Inflammation around cysts disrupting GABAergic pathways in basal ganglia

Anjana KK et al 2020 [34]	India	29/F	15–20 days	Motor and vocal tics	MRI: Multiple ring-enhancing lesions with perilesional edema in bilateral frontal, gangliocapsular, and parietal regions	Psychosis (hallucinations, delusions, disorientation), headache, impaired tandem walking	Not done (family declined)	Albendazole 400 mg BID for 14 days, dexamethasone 4 mg IV TID tapered, mannitol, antiepileptic prophylaxis	Risperidone 2 mg for psychosis and tics	Marked improvement in both psychosis and tics within 1 week	Not available (patient did not return after 1-month visit)	YGTSS score reduced from 55 to 3, MMSE reached 30/30 at 14 days	Likely basal ganglia involvement or edema causing functional disruption in tic-regulating circuits

Campos EM et al 2018 [35]	Ecuador	21/F	Not clearly specified (known NCC for 2 years)	Cerebellar outflow tremor, ophthalmoparesis	Racemose/extraparenchymal NCC; MRI: intraventricular cysts (right lateral and fourth ventricle), communicating hydrocephalus with transependymal CSF egress	Bilateral Babinski signs	Opening pressure 16 cm H2O; other CSF findings not detailed	Steroids and Albendazole for 3 weeks	Same as antiparasitic and anti-inflammatory therapy	Marked clinical and radiological improvement within 3 weeks	Post-treatment MRI showed resolution of hydrocephalus and cysts	Reversal of tremor and ophthalmoparesis	Hydrocephalus and cyst-related pressure or inflammatory changes affecting cerebellar and cranial nerve pathways

Yoganathan et al 2016 [36]	India	11/M	1 month	Perioral dyskinesia, dystonia	Single ring-enhancing granuloma (right posterior temporal lobe), hyperintensities in basal ganglia, thalami, SN, hippocampi	Fever, headache, seizures, altered sensorium, hemiparesis	Not provided in table, diagnosis based on serology and imaging	Albendazole 15 mg/kg/d × 28d + Prednisolone 1 mg/kg/d × 5d	Antispasticity drugs, supportive care	Marked improvement in sensorium and dystonia	Resolution of signal changes; granuloma persisted with reduced edema; no calcification	Residual hemiparesis and dystonia; good ambulation	JE involvement of basal ganglia in patient with preexisting NCC

		13/M	3 months	Meige syndrome, generalized dystonia, oromandibular dystonia, blepharospasm, bradykinesia	Multiple ring-enhancing cysticercal granulomas (right cingulate and inferior frontal gyri); gyral hyperintensities and basal ganglia involvement	Extrapyramidal symptoms, rigidity, hypophonia, difficulty swallowing	Normal initially; serology positive for JE	Ribavirin + Clonazepam, tetrabenazine, trihexyphenidyl	Symptomatic drugs for dystonia and dystonic spasms	Significant reduction in oromandibular and hand dystonia	Resolution of hyperintensities, persistent ring enhancement of granulomas, calcified scolex on CT	Marked clinical improvement	Basal ganglia damage by JE in presence of NCC may contribute to movement disorders

Gokhale et al 2015 [37]	India	8/M	7 days	Focal myoclonus (right upper and lower limbs)	MRI: Solitary lesion in left frontal lobe with perilesional edema, consistent with NCC	Brisk DTRs, bilateral ankle clonus, no other focal deficits	Not done (other metabolic, autoimmune, Wilson’s disease ruled out)	Albendazole 15 mg/kg/day, oral prednisolone 2 mg/kg/day for 5 days	Same as antiparasitic/steroid therapy	Marked improvement within 2 days; complete resolution of myoclonus	Not reported	Full clinical recovery with discharge	Inflammatory edema in frontal cortex causing focal cortical myoclonus via sensorimotor hyperexcitability

Venkatarathna mma et al 2013 [38]	India	25/M	1 week	Cerebellar ataxia, Orofacial dyskinesia, Choreiform movements	Disseminated neurocysticercosis with multiple lesions in cerebral and cerebellar hemispheres, brainstem, spinal cord, intramuscular and subcutaneous tissue	Scanning dysarthria, horizontal gaze nystagmus, bilateral cerebellar signs	Not reported	Albendazole, corticosteroids	Discontinuation of phenytoin; switched to sodium valproate	Significant improvement; symptoms resolved within 3 days	Not specifically reported	Symptomatic improvement, regular follow-up	Phenytoin toxicity; cerebellar ataxia also possible due to neurocysticercosis

Karnik et al 2011 [39]	India	11/F	5 years	Right hemiballismus	Solitary ring-enhancing lesion in the left thalamus on MRI suggestive of NCC	Otherwise normal neurological examination	Not detailed; serological and autoimmune workup negative	Albendazole + prednisolone	Haloperidol initially (no response); main treatment was antiparasitic and steroids	Marked improvement within 2 days of starting antiparasitic/steroid therapy	Planned but patient lost to follow-up	Dramatic symptomatic improvement; long-standing hemiballismus resolved	Lesion in the thalamus, a structure involved in motor control, likely caused involuntary flinging movements

Dewan et al 2011 [40]	India	10/F	4 days	Chorea (generalized choreiform movements)	MRI: Ring-enhancing lesion (5.2 × 4.8 mm) in right paramedian midbrain with mild perilesional oedema (suggestive of active NCC)	Quadriparesis (power 3/5–4/5), sluggish reflexes, extensor plantar response, slurred speech, emotional lability	Normal blood counts, ESR, CRP, ASO titre, ANA, ceruloplasmin; no CSF analysis reported	Albendazole (started after steroids); steroids; mannitol for raised ICP	Haloperidol	Dramatic improvement in chorea within 2 weeks; returned to school at 3 months	Not performed	Good recovery without neurological deficit	Midbrain NCC lesion affecting extrapyramidal pathways

Razdan et al 2009 [41]	India	20/M	1 week	Hemifacial spasm (right)	MRI: Isointense ring lesion with eccentric scolex and perilesional edema in right posterior pons at facial/acoustic nerve exit	Headache, vertigo, imbalance, fever, recurrent vomiting (resolved); facial twitching persisted	Peripheral eosinophilia; CSF not done	Albendazole (8 days), prednisolone (14 days)	Not separately given	Complete resolution of symptoms	Follow-up MRI not done due to affordability	Full recovery	Parenchymal lesion near facial nerve nucleus in pons causing irritation or compression of the nerve

Bhatia R et al 2008 [42]	India	45/M	2 weeks	Isolated facial myokymia (right side)	MRI: Ring-enhancing lesion with scolex and edema in right pons (colloidal-vesicular stage)	Normal cranial nerves except facial myokymia; no limb weakness or cerebellar signs	CSF: Normal protein and sugar; no pleocytosis; negative for organisms and TB-PCR	Oral prednisolone tapered over 2 weeks; no antiparasitic given	Clonazepam (initial), then switched to carbamazepine due to incomplete response	Marked improvement with carbamazepine; complete resolution in 2 months	Follow-up MRI: Decrease in lesion size and edema	Complete resolution of symptoms; no recurrence	Pontine lesion near facial nerve nucleus likely caused hyperexcitability of facial motor neurons

Hamed and El-Metaal 2006 [43]	Egypt	21/F	2 years	Left hemichorea	CT: Single calcified lesion in right caudate nucleus	No other focal neurological deficits reported	CSF: Normal (non-inflammatory); serology for cysticercosis positive in serum	Prednisolone (short course); no antiparasitic needed for calcified stage	Haloperidol 1 mg/day	Marked improvement in chorea within a few days; full remission at 4 weeks	CT confirmed persistent calcification; no active lesion	Symptom-free at 8-month follow-up	Calcified NCC in basal ganglia disrupting motor circuits, leading to hemichorea

Verma et al 2006 [44]	India	12/F	Acute onset	Right hemichorea	CT: Single ring-enhancing lesion with perifocal edema in left thalamic area suggestive of NCC	No specific additional findings reported	Not reported	Albendazole and steroids	Haloperidol	Marked improvement at 1 month	Repeat CT showed resolution of lesion	Complete recovery	Thalamic lesion disrupting motor circuits involved in contralateral movement regulation

Cosentino C, et al 2006 [45]	Peru	22/F	2.5 years	Left hemichorea with foot dystonia and episodic facial deviation	CT: Multiple viable cysts and calcified scars, some with scolex; lesions in cortex, subcortex, and basal ganglia (putamen)	Generalized hypotonia, hyperreflexia, episodic facial asymmetry	Normal hemogram; positive serum Western blot for Taenia solium; stool negative for parasites	Albendazole (15 mg/kg/day × 30 days), Dexamethasone (up to 16 mg/day), Phenytoin	None specifically mentioned; managed with anti-parasitic and steroids	Marked improvement starting with steroids; complete remission over time	CT after 6 months: minimal decrease in viable cysts	Full clinical remission; one partial complex seizure post-treatment	Likely due to inflammation surrounding cysts in basal ganglia; not cyst death itself

Bouldin and Pinter 2006 [46]	USA	11/M	Acute onset; recurrent episodes over 2 days	Transient left hemichorea	Periarterial NCC; MRI showed T2 hyperintensity near right MCA, gadolinium enhancement; MRA showed M1 segment stenosis	None during evaluation; episodes limited to movement abnormalities	WBC: 122/mm^3^ (87% lymphocytes), protein 35 mg/dL, glucose 64 mg/dL, negative cytology, cultures, AFB; CSF ELISA negative	Prednisone (60 mg/day for 2 weeks, then taper), aspirin (81 mg/day); antiparasitic not given due to infarct risk	Supportive; no specific anti-chorea medication	No recurrence of chorea; clinical improvement	MRI and MRA at 6 weeks showed resolution of vessel stenosis and edema	No recurrence at 28 months	Ischemia in basal ganglia due to inflammatory vasculitis/stenosis of right MCA from periarterial NCC

Scott et al 2005 [47]	India	1/M	4 days	Involuntary movements (tremors) involving tongue, left upper and lower limbs	CT: Multiple ring-enhancing lesions in cortical, subcortical and basal ganglia regions	Fever, inability to stand/walk, left-sided focal seizures developed later	Traumatic tap: WBC 70 (90% lymphocytes), RBCs 1250, protein 41 mg/dL, glucose 67 mg/dL	Albendazole with steroid cover	Haloperidol not mentioned; improvement noted with antiparasitic and steroid therapy	Gradual improvement; no abnormal movements at discharge	Not mentioned	Seizure-free and well at 3-month follow-up	Basal ganglia involvement by NCC causing involuntary movements; likely due to lesion-related inflammation

Psarros et al 2003 [48]	USA	26/F	18 months	Akinetic mutism	Cystic lesion at left foramen of Monro; hydrocephalus on CT and MRI	Papilledema; blurred vision; lethargy	Positive for cysticercosis antibodies	Albendazole (postoperatively)	Bromocriptine 25 mg/day	Marked improvement; full recovery within 26 days	CT and MRI at 6 months: no recurrence, normalized ventricles	Complete recovery	Disruption of ascending dopaminergic pathways during surgery; intraventricular hemorrhage; transient hydrocephalus

Gutierrez et al., 1998 [49]	Mexico	69/F	7 months (headache, hearing issues, confusion); hemifacial spasm transient post-surgery	Left hemifacial spasm	Subarachnoid cyst rostral to brainstem, compression of pons, calcifications in basal cisterns and right temporal lobe, hydrocephalus	Confusion, bilateral papilledema, upward gaze paresis, impaired hearing (left), brisk reflexes, bilateral Babinski	19 WBC/mm^3^ (100% lymphocytes), protein 45 mg/dL, glucose 48 mg/dL, positive ELISA for cysticercus antibodies	Prednisone 50 mg orally thrice weekly; no cysticidal drugs	Not specific; supportive management with CSF shunting and steroids	Hemifacial spasm resolved in 3 months	MRI at 58 months: reduction of cyst, no abnormal enhancement	Complete resolution of hemifacial spasm, no recurrence at 5-year follow-up	Compression of facial nerve root exit zone by cyst; altered brainstem dynamics post-CSF shunt

Keane JR, 1995 [50]	USA	32/F	Several recurrences over 14 months	Severe resting and postural tremor (head, jaw, tongue, right upper extremity)	Obstructive hydrocephalus from NCC; mild dilation of third ventricle	Pretectal signs, convergence nystagmus, stupor	Not detailed	Shunt revision, low-pressure valve	Shunt revision	Marked improvement with shunt revision; tremor disappeared	Not detailed	Symptom-free after final shunt	Raised intracranial pressure due to shunt obstruction; possible basal ganglia involvement

Beydoun et al 1994 [51]	USA	34/M	Several years (recurrent hydrocephalus); facial myokymia noted later	Facial myokymia (right orbicularis oris, mentalis, orbicularis oculi)	Multiple lobulated cysts in aqueduct, 4th ventricle, supracerebellar, interpeduncular, ambient, and prepontine cisterns	Tremor (bilateral upper limbs), diplopia, gait instability, bilateral papilledema, upward gaze paresis	Not reported in detail	Praziquantel, Decadron (steroids), Dilantin	Supportive with above treatment	Modest improvement in myokymia and neurological symptoms	MRI showed cystic lesions; no follow-up scan detail given	Partial clinical improvement	Compression and/or inflammation of facial nerve by subarachnoid cysts, toxic or hypoxic effects

Puri et al 1991 [52]	India	11/F	1 month	Myoclonus (generalized, tactile-sensitive)	Multiple cystic lesions with peripheral enhancement and meningeal inflammation; diffuse low-density lesions on CT	Generalized EEG discharges; no other focal neurological deficits	Normal cytology and biochemistry; ELISA positive for cysticercosis	Praziquantel (50 mg/kg/day × 15 days, 2 courses), Sodium valproate	Sodium valproate	Complete resolution of symptoms after second praziquantel course	Normal CT scan post-treatment	Complete recovery	Cortical irritation by active parenchymal cysts; inflammation-induced hyperexcitability

Bhigjee et al 1987 [53]	South Africa	15/F	2 months	Hemichorea (left-sided)	CT scan: Multiple cysts with calcification and contrast enhancement, especially in basal ganglia (right caudate, internal capsule, lenticular nucleus)	Left-sided facial distortions, non-stereotyped jerks, flinging of left arm and leg; no other systemic abnormalities	Positive cysticercus haemagglutination and fluorescent antibody test (titres 1:16 and 1:1 respectively); no eosinophils	Haloperidol; no antiparasitic used	Haloperidol	Some improvement noted	Not available (patient lost to follow-up)	Partial improvement, long-term outcome unknown	Cystic lesions in basal ganglia causing contralateral hemichorea


ANA = Antinuclear Antibody; ASO = Anti-Streptolysin O; BID = Bis in Die (Twice Daily); CSF = Cerebrospinal Fluid; CT = Computed Tomography; DTR = Deep Tendon Reflex; EEG = Electroencephalography; ELISA = Enzyme-Linked Immunosorbent Assay; ESR = Erythrocyte Sedimentation Rate; IIF = Indirect Immunofluorescence; IV = Intravenous; JE = Japanese Encephalitis; MMSE = Mini-Mental State Examination; MRI = Magnetic Resonance Imaging; MRA = Magnetic Resonance Angiography; NA = Not Available/Not Applicable; NCC = Neurocysticercosis; SN = Substantia Nigra; VP Shunt/VPS = Ventriculoperitoneal Shunt; WBC = White Blood Cell Count; YGTSS = Yale Global Tic Severity Scale.

The mean age in the hypokinetic group was 39.3 years (range 6–69 years), while the hyperkinetic group was younger with a mean age of 25.8 years (range 1 to 77 years). Male patients slightly outnumbered females in both groups, comprising 52.38% of the hypokinetic group and 54.17% of the hyperkinetic group (Table 3).

**Table 3:** Spectrum of hypokinetic and hyperkinetic movement disorders in neurocysticercosis.


CHARACTERISTICS	HYPOKINETIC MOVEMENT DISORDERS (N = 21)	HYPERKINETIC MOVEMENT DISORDERS (N = 24)

**Age (in years)**	Mean: 39.3 Median: 38 Mode: 38, 60 Range: 6–69 Interquartile range: 35	Mean: 25.8 Median: 21 Mode: 11 Range: 1–77 Interquartile range: 22

**Sex**	Male (M): 11 (52.38%) Female (F): 10 (47.62%)	Male (M): 13 (54.17%) Female (F): 11 (45.83%)

**Geographical areas of published records**	India: 7 (43.75%) Brazil: 4 (25.0%) Spain: 2 (12.5%) Madagascar: 1 (6.25%) Ecuador: 1 (6.25%) USA: 1 (6.25%) **Total = 16**	India: 11 (52.38%) USA: 4 (19.05%) Peru: 2 (9.52%) Portugal: 1 (4.76%) Ecuador: 1 (4.76%) Egypt: 1 (4.76%) Mexico: 1 (4.76%) **Total = 21**

**Type of movement disorder**	Parkinsonism (all types combined): 11 (52.38%) PSP-like syndrome: 1 (4.76%) Dystonia (all types): 5 (23.81%) Abnormal limb posturing): 3 (14.29%) Other (Mutism, Echolalia, Gegenhalten, Cataplexy: 1 (4.76%)	Chorea/Hemichorea/Hemiballismus: 7 (29.17%) Myoclonus: 4 (16.67%) Tremor (Cerebellar/Resting/Postural): 3 (12.50%) Facial Dyskinesias/Hemifacial Spasm/Myokymia: 6 (25.00%) Dyskinesia (Oromandibular/Focal/Generalized): 4 (16.67%) Other Hyperkinetic/Mixed/Complex Syndromes: 2 (8.33%)

**Other neurological findings**	Cognitive & Behavioral Abnormalities: 6 (28.57%) Cranial Nerve Involvement & Ocular Abnormalities: 6 (28.57%) Motor Weakness & Pyramidal Signs: 6 (28.57%) Gait Disturbance & Postural Instability: 6 (28.57%) Seizures & Epilepsy: 3 (14.29%) Signs of Raised ICP: 4 (19.05%)	Signs of Raised ICP: 5 (20.83%) Pyramidal/Motor Weakness/Reflex Changes: 6 (25.00%) Cerebellar/Brainstem Signs: 4 (16.67%) Seizures/Epilepsy/Psychosis/Encephalopathy: 4 (16.67%) Normal/No Significant Additional Neurological Findings: 6 (25.00%)

**Type of Neurocysticercosis/ neuroimaging**	Parenchymal NCC: 8 (38.10%) Intraventricular NCC: 6 (28.57%) Thalamic/Midbrain/Brainstem NCC: 6 (28.57%) Subarachnoid/Racemose NCC: 2 (9.52%)	Parenchymal NCC (multiple lesions or calcifications): 9 (37.50%) Solitary Ring-Enhancing Granuloma: 2 (8.33%) Extraparenchymal (Intraventricular or Racemose) NCC: 7 (29.17%) Mixed Parenchymal + Extraparenchymal NCC: 3 (12.50%) Subarachnoid/Cistern NCC (e.g., CPA, basal cisterns): 2 (8.33%) Periarterial/Vascular NCC: 1 (4.17%) Disseminated/Multisystem NCC: 1 (4.17%)

**Duration of illness**	<1 month: 5 (26.32%) 1–6 months: 4 (21.05%) >6 months – 1 year: 2 (10.53%) >1 year – 5 years: 4 (21.05%) >5 years: 4 (21.05%) **Not clearly stated**: 3 (13.64%)	<1 month: 5 (20.83%) 1–6 months: 6 (25.00%) >6 months – 1 year: 2 (8.33%) >1 year – 5 years: 5 (20.83%) >5 years: 3 (12.50%) Not clearly stated: 3 (12.50%)

**CSF Abnormalities**	Positive Serology for NCC: 9 (42.86%) Inflammatory CSF (pleocytosis, high protein): 5 (23.81%) Normal CSF: 3 (14.29%) Not Reported: 6 (28.57%) Serology Negative: 1 (4.76%)	CSF Positive for Cysticercosis: 6 (25.00%) Normal or Non-Inflammatory CSF: 4 (16.67%) Inflammatory CSF: 3 (12.50%) **CSF Not Done/Not Reported:** 11 (45.83%)

**Treatment Given Antiparasitic/Steroids)**	Albendazole Given: 14 (66.67%) Praziquantel Given: 2 (9.52%) Steroids Administered: 13 (61.90%) No Antiparasitic/Deferred: 3 (14.29%) Surgical Intervention: 4 (19.05%) Not Specified/NA: 3 (14.29%)	Albendazole + Steroids: 13 (54.17%) Albendazole Alone or Given (Steroid status unclear): 2 (8.33%) Steroids Alone (No antiparasitic): 3 (12.50%) Praziquantel-Based Therapy: 2 (8.33%) No Antiparasitic/Symptomatic Only: 2 (8.33%) Other Symptomatic/Adjuvant Therapy: 2 (8.33%)

**Treatment of Movement Disorder**	Levodopa-Based Therapy: 6 (28.57%) Anticholinergic Agents: 3 (14.29%) Benzodiazepines/Sedatives: 2 (9.52%) Dopaminergic/Other Parkinsonian Support (e.g., Amantadine): 1 (4.76%) VP Shunt/Surgical Management: 2 (9.52%) Botulinum Toxin/Dystonia-Specific Therapy: 1 (4.76%) Physiotherapy/Supportive Only: 2 (9.52%) Anticonvulsants: 1 (4.76%) None Given/Not Mentioned: 7 (33.33%)	Antipsychotic Agents (Haloperidol, Risperidone): 7 (29.17%) Antiepileptics: 5 (20.83%) Surgical Intervention/CSF Shunting: 3 (12.50%) Other Movement Disorder Medications: 3 (12.50%) Supportive/Not Specifically Mentioned: 6 (25.00%)

**Response to Treatment**	Marked or Complete Clinical Improvement: 10 (47.62%) Partial or Gradual Improvement: 5 (23.81%) No Improvement/Clinical Deterioration: 3 (14.29%) Radiological Improvement Only: 1 (4.76%) NA: 2 (9.52%)	Complete Resolution/Full Recovery: 9 (37.50%) Marked Improvement: 11 (45.83%) Gradual or Delayed Improvement: 2 (8.33%) Partial/Some Improvement: 1 (4.17%) Outcome Not Clearly Specified: 1 (4.17%)

**Follow-up Neuroimaging, if any**	Lesion Resolution/Improvement: 3 (14.29%) Calcification/Residual Sequelae: 2 (9.52%) MRI/CT Changes Without Clear Outcome: 3 (14.29%) NA: 13 (61.9%)	Lesion/Cyst Resolution: 7 (29.17%) Partial Resolution/Persistent Findings: 4 (16.67%) Calcification/Sequelae Without Active Disease: 2 (8.33%) Not Performed/Not Available/Lost to Follow-Up: 8 (33.33%) Intraoperative or Non-standard Imaging Only: 1 (4.17%) Normal Imaging Post-Treatment: 2 (8.33%)

**Outcome**	Complete/Full Recovery: 6 (28.57%) Improved/Marked Clinical Improvement: 6 (28.57%) Partial Improvement/Persistent Symptoms: 3 (14.29%) Persistent Disability: 1 (4.76%) Death: 2 (9.52%) NA: 2 (9.52%)	Complete Recovery/Full Remission: 9 (37.50%) Marked/Dramatic Improvement: 6 (25.00%) No Recurrence/Seizure-Free/Stable at Follow-Up: 4 (16.67%) Partial Improvement/Residual Symptoms: 3 (12.50%) Objective Functional Scores Reported: 2 (8.33%)

**Possible reasons of movement disorders**	Basal Ganglia Involvement: 11 (52.38%) Midbrain/Brainstem Involvement: 10 (47.62%) Thalamic/Cerebello-Thalamo-Cortical Pathway Involvement: 5 (23.81%) Cortico-Subcortical Motor Circuit Disruption: 2 (9.52%) Hydrocephalus/Raised ICP Effects: 6 (28.57%) Inflammatory/Immune-Mediated Pathogenesis: 8 (38.10%) Epileptogenic Spread (Ictal Dystonia): 1 (4.76%)	Basal Ganglia Involvement: 10 (41.67%) Thalamic/Subthalamic Involvement: 3 (12.50%) Facial Nerve Involvement (Nucleus or Exit Zone): 5 (20.83%) Cortical Hyperexcitability/Motor Cortex Involvement: 3 (12.50%) Brainstem/Midbrain Extrapyramidal Pathway Disruption: 2 (8.33%) Hydrocephalus/CSF Dynamic Alteration/Raised ICP: 4 (16.67%) Drug-Induced/Toxic Etiology: 1 (4.17%)


AFB = Acid-Fast Bacilli; Alb = Albendazole; CPA = Cerebellopontine Angle; CSF = Cerebrospinal Fluid; CT = Computed Tomography; ICP = Intracranial Pressure; MRI = Magnetic Resonance Imaging; NA = Not Available; NCC = Neurocysticercosis; PSP = Progressive Supranuclear Palsy; VP = Ventriculoperitoneal.

Most of the reported cases originated from India. In the hypokinetic group, 52.38% of reports originated from India, while in the hyperkinetic group, India contributed 43.75% of the cases. Other countries included the United States, Brazil, Peru, Spain, Madagascar, Portugal, Ecuador, Egypt, and Mexico. The duration of illness varied widely across both groups. In the hypokinetic group, 26.32% presented within one month of symptom onset, while 21.05% had symptoms persisting beyond five years. A similar pattern was seen in the hyperkinetic group, where 20.83% presented acutely and another 33.3% had chronic symptoms of over one year (Table 3).

Among the hypokinetic group, parkinsonism was the most common presentation, observed in 52.38% of cases. One patient presented with features mimicking progressive supranuclear palsy. Dystonia and abnormal limb posturing were also noted. In contrast, the hyperkinetic group demonstrated greater diversity: chorea or hemichorea occurred in 29.17% of patients, facial dyskinesias or hemifacial spasms in 25%, myoclonus in 16.67%, and tremors in 12.5%. Oromandibular and generalized dyskinesias were also seen in 16.67% of hyperkinetic cases. (Video) Additional neurological symptoms were common in both groups. Regarding other associated neurological features, 28.57% of patients in the hypokinetic group exhibited cognitive or behavioral disturbances, cranial nerve involvement, pyramidal signs, gait abnormalities, or seizures. In the hyperkinetic group, 25% presented with pyramidal or reflex abnormalities, 20.83% showed signs indicative of raised intracranial pressure, and 16.67% demonstrated either cerebellar signs or neuropsychiatric manifestations, including psychosis or encephalopathy (Table 3). Out of a total of 45 patients, 7 patients (15.6%) had both hypokinetic and hyperkinetic movement disorders. The most common pattern observed was parkinsonism with bradykinesia, rigidity, and tremor, seen in two cases.

**Video:** Hemichorea in a patient with neurocysticercosis. The video demonstrates continuous, irregular, non-rhythmic involuntary movements predominantly affecting the right upper limb. These hyperkinetic movements are consistent with hemichorea. Neuroimaging revealed a cystic lesion with an eccentric scolex and surrounding edema. Written informed consent was obtained from the patient for video recording and publication.

**Figure 2:**
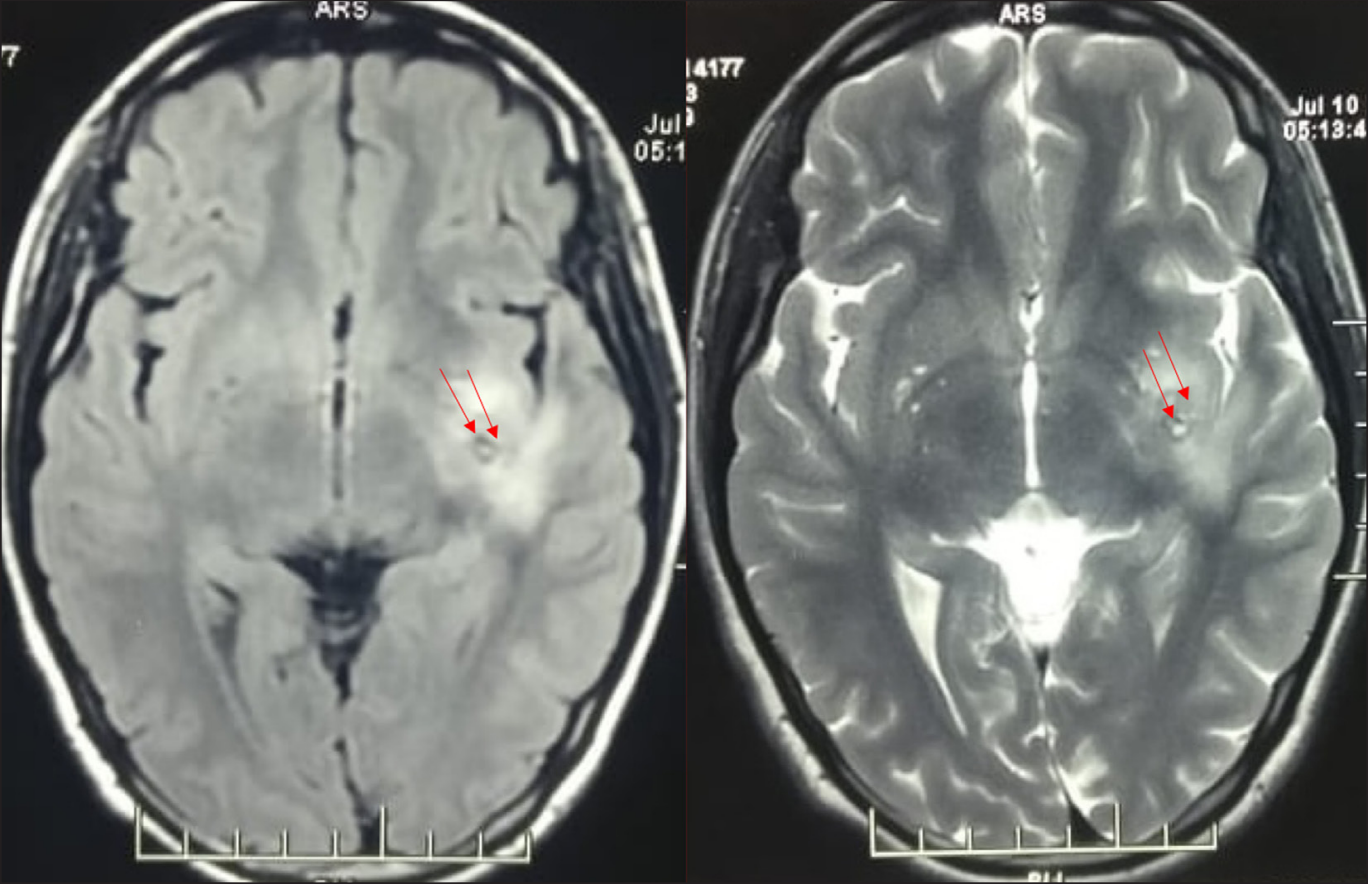
MRI of the brain reveals a cystic lesion with an eccentric scolex and surrounding edema located near left thalamic region. Clinically, the patient presented with right-sided hemichorea (Video).

Parenchymal neurocysticercosis was the most common form, seen in approximately 38% of both hypokinetic and hyperkinetic groups. Intraventricular or racemose types were observed in about 28–29% across both groups. Thalamic, brainstem, and midbrain involvement was more frequent in hypokinetic cases (28.57%). (Figure 2) Less common forms included subarachnoid cysts, solitary granulomas, and mixed patterns. In the hypokinetic group, 42.86% of patients had serological evidence of neurocysticercosis, and 23.81% showed inflammatory cerebrospinal fluid findings. In the hyperkinetic group, serology was positive in 25%, while 45.83% had either no cerebrospinal fluid analysis or it was not reported. Inflammatory cerebrospinal fluid features were present in 12.5% of cases (Table 3).

Albendazole was the primary antiparasitic therapy in both groups, administered in 66.67% of hypokinetic and 54.17% of hyperkinetic patients. Corticosteroids were co-administered in 61.9% and 54.17%, respectively. Praziquantel-based therapies were used less frequently. Surgical interventions, such as ventriculoperitoneal shunting, were reported in 19.05% of hypokinetic and 12.5% of hyperkinetic cases, often in the context of obstructive hydrocephalus or large intraventricular cysts. In the hypokinetic group, movement disorder-specific treatments included levodopa (28.57%), anticholinergics (14.29%), and dopaminergic agents such as amantadine (4.76%). Supportive therapies like physiotherapy and sedatives were used in selected cases. In the hyperkinetic group, antipsychotic drugs (29.17%), antiepileptics (20.83%), and botulinum toxin were utilized, with varying results. A subset of patients received only symptomatic or supportive care (Table 3).

Treatment outcomes were generally favorable. In the hypokinetic group, about 71% showed clinical improvement, while 14.29% had no recovery or worsened. In the hyperkinetic group, over 83% improved, and only 12.5% had residual symptoms. Lesion resolution on follow-up imaging was seen in 14.29% of hypokinetic and 29.17% of hyperkinetic cases, though repeat imaging was often missing. At final follow-up, 29–38% achieved full recovery, and 25–30% showed marked improvement. Persistent deficits were more common in hypokinetic patients, with two deaths reported in this group (Table 3).

Basal ganglia involvement was considered the most common mechanism of movement disorders in both groups (hypokinetic: 52.38%; hyperkinetic: 41.67%). Midbrain and brainstem involvement was particularly common in hypokinetic patients. Thalamic and cerebellar-thalamo-cortical pathway lesions, hydrocephalus with raised intracranial pressure, immune-mediated inflammation and cortical hyperexcitability were additional proposed mechanisms. In one case, ictal dystonia was attributed to epileptogenic spread. A drug-induced etiology (phenytoin toxicity) was considered in a hyperkinetic patient with a history of multiple medications. Patient improved after withdrawal of phenytoin (Table 3).

In a 20-year descriptive cohort study from the Neurocysticercosis Registry at Ecuador, 23 out of 590 patients developed movement disorders. The spectrum included parkinsonism (15 cases), tremor (5), dystonia (2), and chorea (1). Parkinsonism was more common in middle-aged patients with widespread subarachnoid or ventricular cysts, while dystonia and chorea occurred in younger females with basal ganglia lesions. Prognosis varied by disorder: all patients with chorea, dystonia, and tremor fully recovered, whereas nearly half of those with parkinsonism required prolonged treatment, including surgery and long-term levodopa therapy, indicating a relatively worse outcome [7].

## Discussion

Neurocysticercosis is associated with a remarkably broad and heterogeneous range of movement disorders, underscoring its potential to affect multiple motor control regions within the central nervous system. This systematic review identified both hypokinetic and hyperkinetic manifestations across varying age groups, clinical contexts, and anatomical sites. Among hypokinetic disorders, parkinsonism emerged as the most frequent presentation, followed by bradykinesia and rigidity—often resembling idiopathic parkinsonian syndromes and typically associated with lesions in the basal ganglia or midbrain. Conversely, hyperkinetic manifestations were more varied, including chorea or hemichorea, hemifacial spasm, facial dyskinesias, and myoclonus. Less common but noteworthy presentations included dystonia, tremor, ballism, and asterixis. Several cases involved overlapping movement abnormalities, suggesting multifocal central nervous system involvement. This wide clinical spectrum reflects the ability of neurocysticercosis to imitate various extrapyramidal disorders, frequently with acute or subacute onset and variable clinical outcomes based on factors such as lesion location, patient age, and promptness of therapy.

The pathogenesis of movement disorders in neurocysticercosis is multifactorial and depends on the location, stage, and type of the cystic lesion. Parenchymal neurocysticercosis, particularly in the basal ganglia, midbrain, or thalamus, is commonly associated with hypokinetic disorders such as parkinsonism. In these cases, cystic or granulomatous inflammation may disrupt the nigrostriatal dopaminergic pathway, leading to bradykinesia, rigidity, and tremor. Inflammatory edema or immune-mediated neuronal dysfunction—rather than irreversible damage—is likely responsible in some cases, explaining the partial or complete response to antiparasitic and steroid therapy. Hyperkinetic movement disorders, on the other hand, are more often associated with subthalamic nucleus, thalamic, or caudate lesions, which may produce chorea, dystonia, or ballism due to disinhibition of thalamocortical pathways. Intraventricular or subarachnoid cysts can lead to hydrocephalus or raised intracranial pressure, secondarily affecting motor circuits. Experimental studies show that larval products directly excite neurons by releasing glutamate and aspartate, activating glutamate receptors and inducing seizure-like activity, suggesting a role in abnormal brain signalling and seizures [54]. In some cases, movement disorders may also arise from cortical irritation, epileptogenic spread, or post-infectious immune responses. Thus, the type of neurocysticercosis—parenchymal, extraparenchymal, intraventricular, or mixed—determines the pathophysiological mechanism and clinical presentation [55,56,57].

Antiparasitic therapy, particularly albendazole, is the mainstay of treatment for movement disorders associated with neurocysticercosis, especially in patients with active or parenchymal cystic lesions. Albendazole is typically administered at a dose of 15 mg/kg/day generally for 2 to 4 weeks. In approximately 65% of reviewed cases, it was used either alone or in combination with corticosteroids. Concomitant corticosteroids help attenuate the host inflammatory response to degenerating cysticerci, reduce perilesional edema, and prevent clinical worsening during treatment [58]. Hyperkinetic movement disorders, such as hemichorea, hemiballismus, and myoclonus, generally responded rapidly and favorably to antiparasitic therapy, often resolving within days to weeks—particularly when the lesions were localized to areas such as the thalamus or motor cortex and detected in the early inflammatory phase. In contrast, hypokinetic disorders, particularly parkinsonism, showed a more variable and often prolonged clinical course. While some patients improved with adjunctive dopaminergic therapy, especially in the presence of reversible hydrocephalus or midbrain involvement, others experienced persistent or worsening symptoms due to irreversible neuronal damage, delayed diagnosis, or complications such as shunt malfunction or extensive brainstem involvement. In a reported series of 23 patients with movement disorders due to neurocysticercosis, most achieved complete recovery following antiparasitic treatment without requiring long-term therapy. However, parkinsonism cases were more complex, often necessitating multiple interventions. Seven patients required repeat courses of albendazole with corticosteroids, eight underwent cyst excision, and eight required ventriculoperitoneal shunting. Twelve patients received levodopa, though its benefits were limited, particularly in severe cases. Long-term neurological outcomes ranged widely, with some patients achieving full recovery and others remaining with significant deficits, often accompanied by complications such as epilepsy, cognitive decline, and intracranial hypertension [7].

Symptomatic treatment is essential, particularly in movement disorders. Hypokinetic patients with parkinsonism may benefit from dopaminergic therapy, such as levodopa, especially when reversible disruption of the nigrostriatal pathway is suspected. Hyperkinetic disorders require individualized approaches—antipsychotics for chorea, benzodiazepines or valproate for myoclonus, and botulinum toxin for focal dystonias or hemifacial spasm. In cases with raised intracranial pressure due to intraventricular cysts, neurosurgical intervention like ventriculoperitoneal shunting may be necessary [59]. Most patients respond well to antiparasitic treatment, with over 80% achieving partial or complete recovery, though delayed diagnosis or calcified lesions may result in persistent symptoms or incomplete resolution [60].

This review is limited by the inclusion of only case reports, case series, and observational studies, leading to potential selection and publication bias. Diagnostic criteria were inconsistent, follow-up imaging was often lacking, and treatment protocols varied. Additionally, causality between neurocysticercosis and movement disorders could not be firmly established in all cases.

In conclusion, neurocysticercosis can cause a wide range of hypokinetic and hyperkinetic movement disorders, often with reversible symptoms if diagnosed and treated early. Timely neuroimaging, appropriate antiparasitic and symptomatic therapy, and multidisciplinary care are key to favorable outcomes.

## Data Accessibility Statement

All data generated or analyzed during this study are provided in the published article and its supplementary materials.

## Additional File

The additional file for this article can be found as follows:

10.5334/tohm.1061.s1 Supplementary Material. Item-1 to Item-2.
