# Supplementary material for: Movement Disorders in Neurocysticercosis: A Systematic Review
Source: Tremor Other Hyperkinet Mov (N Y). 2025 Aug 8;15:38. doi: 10.5334/tohm.1061 (PMC12352383; doi:10.5334/tohm.1061)
Supplement: Supplementary Material. — Item-1 to Item-2. [file tohm-15-1-1061-s1.zip › tohm-1061_garg-s1/688d69620ed9d.docx]

**Movement Disorders in Neurocysticercosis: A Systematic Review of Case Reports, Case Series, and Cohort Studies**

**Evaluation of the methodological quality of case reports and case series (n = 45)**

| **Reference** | **Does the patient represent the whole experience of the investigator** | **Was the exposure adequately ascertained?** | **Was the outcome adequately ascertained?** | **Were other alternative causes that may explain the observation ruled out?** | **Was there a challenge and/or re-challenge phenomenon?** | **Was there a dose-response effect?** | **Was follow-up long enough for outcomes to occur?** | **Is the case(s) described with sufficient details to allow practitioners make inferences related to their own practice?** | **Score** |
| --- | --- | --- | --- | --- | --- | --- | --- | --- | --- |
| **Hypokinetic movement disorders** | | | | | | | | | |
| Das et al 2025 | **Yes** | **Yes** | **Yes** | **Yes** | **NA** | **Yes** | **Yes** | **Yes** | **7** |
| Rasaholiarison et al 2024 | **Yes** | **Yes** | **Yes** | **Yes** | **NA** | **Yes** | **Yes** | **Yes** | **7** |
| Ahmad N et al 2024 | **Yes** | **Yes** | **Yes** | **Yes** | **NA** | **Yes** | **Yes** | **Yes** | **7** |
| Puig et al 2023 | **Yes** | **Yes** | **Yes** | **Yes** | **NA** | **Yes** | **Yes** | **Yes** | **7** |
| Oliveira et al., 2020 | **Yes** | **Yes** | **Yes** | **Yes** | **NA** | **Yes** | **Yes** | **Yes** | **7** |
| Verma R et al., 2013 | **Yes** | **Yes** | **Yes** | **Yes** | **NA** | **Yes** | **Yes** | **Yes** | **7** |
| de Lima PMG et al., 2012 | **Yes** | **Yes** | **Yes** | **Yes** | **NA** | **Yes** | **Yes** | **Yes** | **7** |
| Sharma et al 2011 | **Yes** | **Yes** | **Yes** | **Yes** | **NA** | **Yes** | **Yes** | **Yes** | **7** |
| Prashantha et al 2008 | **Yes** | **Yes** | **Yes** | **Yes** | **NA** | **Yes** | **Yes** | **Yes** | **7** |
| Cabo López et al., 2008 | **Yes** | **Yes** | **Yes** | **Yes** | **NA** | **Yes** | **Yes** | **Yes** | **7** |
| Patel et al 2006 | **Yes** | **Yes** | **Yes** | **Yes** | **NA** | **Yes** | **Yes** | **Yes** | **7** |
|  | **Yes** | **Yes** | **Yes** | **Yes** | **NA** | **Yes** | **Yes** | **Yes** | **7** |
| Sá et al 2005 | **Yes** | **Yes** | **Yes** | **Yes** | **NA** | **Yes** | **Yes** | **Yes** | **7** |
|  | **Yes** | **Yes** | **Yes** | **Yes** | **NA** | **Yes** | **Yes** | **Yes** | **7** |
| Serrano-Dueñas & Placencia, 1999 | **Yes** | **Yes** | **Yes** | **Yes** | **NA** | **Yes** | **Yes** | **Yes** | **7** |
|  | **Yes** | **Yes** | **Yes** | **Yes** | **NA** | **Yes** | **Yes** | **Yes** | **7** |
|  | **Yes** | **Yes** | **Yes** | **Yes** | **NA** | **Yes** | **Yes** | **Yes** | **7** |
|  | **Yes** | **Yes** | **Yes** | **Yes** | **NA** | **Yes** | **Yes** | **Yes** | **7** |
| Sawhney et al., 1998 | **Yes** | **Yes** | **Yes** | **Yes** | **NA** | **Yes** | **Yes** | **Yes** | **7** |
| Verma et al., 1995 | **Yes** | **Yes** | **Yes** | **Yes** | **NA** | **Yes** | **Yes** | **Yes** | **7** |
| De Assis et al., 1955 | **Yes** | **Yes** | **Yes** | **Yes** | **NA** | **Yes** | **Yes** | **Yes** | **7** |
| **Hyperkinetic Movement Disorders** | | | | | | | | | |
| Matos Pereira 2022 | **Yes** | **Yes** | **Yes** | **Yes** | **NA** | **Yes** | **Yes** | **Yes** | **7** |
| Yang et al 2020 | **Yes** | **Yes** | **Yes** | **Yes** | **NA** | **Yes** | **Yes** | **Yes** | **7** |
| Kumar S et al., 2020 | **Yes** | **Yes** | **Yes** | **Yes** | **NA** | **Yes** | **NA** | **NA** | **5** |
| Anjana KK et al., 2020 | **Yes** | **Yes** | **Yes** | **Yes** | **NA** | **Yes** | **Yes** | **Yes** | **7** |
| Campos EM et al., 2018 | **Yes** | **Yes** | **Yes** | **Yes** | **NA** | **Yes** | **Yes** | **Yes** | **7** |
| Yoganathan et al 2016 | **Yes** | **Yes** | **Yes** | **Yes** | **NA** | **Yes** | **Yes** | **Yes** | **7** |
|  | **Yes** | **Yes** | **Yes** | **Yes** | **NA** | **Yes** | **Yes** | **Yes** | **7** |
| Gokhale et al 2015 | **Yes** | **Yes** | **Yes** | **Yes** | **NA** | **Yes** | **Yes** | **Yes** | **7** |
| Venkatarathnamma et al., 2013 | **Yes** | **Yes** | **Yes** | **Yes** | **NA** | **Yes** | **Yes** | **Yes** | **7** |
| Karnik et al 2011 | **Yes** | **Yes** | **Yes** | **Yes** | **NA** | **Yes** | **Yes** | **Yes** | **7** |
| Dewan et al., 2011 | **Yes** | **Yes** | **Yes** | **Yes** | **NA** | **Yes** | **Yes** | **Yes** | **7** |
| Razdan et al 2009 | **Yes** | **Yes** | **Yes** | **Yes** | **NA** | **Yes** | **Yes** | **Yes** | **7** |
| Bhatia R et al 2008 | **Yes** | **Yes** | **Yes** | **Yes** | **NA** | **Yes** | **Yes** | **Yes** | **7** |
| Hamed and El‐Metaal 2006 | **Yes** | **Yes** | **Yes** | **Yes** | **NA** | **Yes** | **Yes** | **Yes** | **7** |
| Verma et al 2006 | **Yes** | **Yes** | **Yes** | **Yes** | **NA** | **Yes** | **Yes** | **Yes** | **7** |
| Cosentino C, et al 2006 | **Yes** | **Yes** | **Yes** | **Yes** | **NA** | **Yes** | **Yes** | **Yes** | **7** |
| Bouldin and Pinter 2006 | **Yes** | **Yes** | **Yes** | **Yes** | **NA** | **Yes** | **Yes** | **Yes** | **7** |
| Scott et al 2005 | **Yes** | **Yes** | **Yes** | **Yes** | **NA** | **Yes** | **Yes** | **Yes** | **7** |
| Psarros et al 2003 | **Yes** | **Yes** | **Yes** | **Yes** | **NA** | **Yes** | **Yes** | **Yes** | **7** |
| Gutierrez et al., 1998 | **Yes** | **Yes** | **Yes** | **Yes** | **NA** | **Yes** | **Yes** | **Yes** | **7** |
| Keane JR, 1995 | **Yes** | **Yes** | **Yes** | **Yes** | **NA** | **Yes** | **Yes** | **Yes** | **7** |
| Beydoun et al., 1994 | **Yes** | **Yes** | **Yes** | **Yes** | **NA** | **Yes** | **Yes** | **Yes** | **7** |
| Puri et al., 1991 | **Yes** | **Yes** | **Yes** | **Yes** | **NA** | **Yes** | **Yes** | **Yes** | **7** |
| Bhigjee et al., 1987 | **Yes** | **Yes** | **Yes** | **Yes** | **NA** | **Yes** | **Yes** | **Yes** | **7** |

**Domains Leading explanatory questions**

**Selection 1. Does the patient(s) represent(s) the whole experience of the investigator or is the selection method unclear to the extent that other patients with similar presentation may not have been reported?**

**Ascertainment 2. Was the exposure adequately ascertained?**

**3. Was the outcome adequately ascertained?**

**Causality**

**4. Were other alternative causes that may explain the observation ruled out?**

**5. Was there a challenge/re-challenge phenomenon?**

**6. Was there a dose–response effect?**

**7. Was follow-up long enough for outcomes to occur?**

**Reporting**

**8. Is the case(s) described with sufficient details to allow other investigators to replicate the research or to allow practitioners make**

**inferences related to their own practice?**
